# An analysis of women’s and children’s health professional requirements in China in 2010 based on workload

**DOI:** 10.1186/s12913-014-0589-y

**Published:** 2014-11-19

**Authors:** Peige Song, Zhenghong Ren, Evropi Theodoratou, Sufang Guo, Lin An

**Affiliations:** Department of Child, Adolescent and Women’s Health, School of Public Health, Peking University, Beijing, China; Centre for Population Health Sciences, The University of Edinburgh, Edinburgh, UK; UNICEF China, Beijing, China

**Keywords:** Women and children’s health, Health professional, China

## Abstract

**Background:**

To make health services more equitable and accessible for women and children and to achieve a universal coverage, human resources for women and children’s health (WCH) should be evaluated. However, since there is still no consensus on the real situation of Chinese WCH professionals, we aim with this study to compare the actual and required amount of WCH professionals for China.

**Methods:**

The data of the actual number of WCH professionals and workload of each service type was obtained by a national institution-based sampling survey. We then estimated the time that a WCH professional spends at work (annually), the time norm of each service schedule and the required number of WCH professionals based on workload. We evaluated the situation of Chinese WCH professionals in 2010 by comparing the actual and required WCH professionals and by calculating the ratios of the actual-to-required number of staff.

**Results:**

There were 515,778 health professionals providing WCH services in the investigated 5,168 medical/health institutions in 2010. Workloads of most WCH services in east areas were larger than that in the central and the west. For women’s health, the numbers of required WCH professionals were 48510, 43992, 40571 and 133073 for the east, the central, the west areas and the whole nation respectively. For children’s health professionals, the corresponding numbers were 56241, 36818, 40618 and 133677 for the east, the central, the west and the whole nation.

**Conclusions:**

The WCH professionals in China were sufficient for workload in 2010, there were still lots of potential capacities to provide better services, especially for women. Strategies should be taken to improve the quality of WCH professionals or their working motivation.

## Background

Women and children’s health (WCH) is regarded as one of the foundations of public health and is critically important for human development [1]. Six of the eight Millennium Development Goals (MDGs) set by the United Nations in 2000 are directly related to women and children’s wellbeing [2]. China is one of the few developing countries set to achieve both MDG 4 (between 1990 and 2015, reduce by two thirds the under-five mortality rate) and 5 (between 1990 and 2015, reduce by three quarters the maternal mortality ratio and achieve universal access to reproductive health) [3,4]. China has achieved this by signing a number of related international conventions, implementing public health service programs and improving the current health services for women and children [5]. However, despite these achievements and successes, there is still progress that needs to be made. In the latest “Outline for the Development of Chinese Women (2011–2020)” and “Outline for the Development of Chinese Children (2011–2020)” [6], the Chinese government presented a plan for the next health developments for women and children, highlighting the need of making health services more equitable and accessible for them and on achieving universal coverage, which means that all women and children can obtain the health services they need without suffering financial hardship when paying for them [7]; a difficult target for many developing countries [8].

In China, women and children receive specialized all-round preventive and curative healthcare by health professionals from general hospitals, WCH institutions and community-based healthcare services at province, prefectural, county, township and village level [1,9]. These services are part of the national maternal and child healthcare system and are defined as WCH professional healthcare. This includes the following services for women: premarital healthcare, prenatal and postnatal care and reproductive healthcare; and healthcare services for infants, young children and pre-school children [5].

The basis of universal health coverage is financial and physical access to necessary good quality health care for all people [10]. In addition to financial and physical resources, the availability of trained health workers can directly affect the performance of the health system [11] and the WHO has emphasized that health workforce shortage has become “the most serious obstacle” in realizing health as human right [12]. There is lack of consensus on the availability of trained health workers in China, especially for those in the maternal and child sectors, with some researchers identifying a surplus and some reporting a shortage [13–15]. It is therefore important to evaluate the situation of Chinese WCH professionals, which will allow the government to plan and prioritize the obstacles that need to be overcome for achieving universal access to health services. There are several methods to estimate health human resource requirements, such as the health human resources density ratio per 10,000 inhabitants, the target-setting approach, the needs-based approach and the utilization approch; all with different advantages and limitations [16,17]. The health human resources density ratio specifies the desired or needed worker-to-population ratio. Although it is a quick and easy to apply and understand method, it provides no information on personnel utilization. In the target-setting approach, targets for the production and delivery of specific outcome oriented health services are set by health authorities and then these targets are converted to health human resource requirements. The needs-based method translates service needs into time estimates assuming that all health needs can and should be met, which are then expressed as full-time person requirements using productivity norms and professional judgment [17]. Neither the target-setting approach nor the needs-based approach takes clients’ perspectives into account. The utilization approach, however, is based on the actual level of use over a year [18], one of the most famous utilization approaches is the Workload Indicators of Staffing Need (WISN) method, it’s a workload-based human resource tool developed by the World Health Organization (WHO) and has been used in many countries or regions to estimate human resources needs [19,20]. With this method, the difference between the actual and required number of health workers and the workload pressure of health workers can be calculated. However, this method needs very clear boundaries of health worker type and facility type, which is almost impossible for a large developing country like China, where the professional responsibilities of doctors, nurses and midwifes is still not strictly classified across the whole country [21,22]. So, in this study, we apply the utilization approach to estimate the required number of WCH professionals based on the actual workload in 2010, and then evaluate the WCH stock appropriateness at that year by calculating the difference between actual and required WCH professionals and the actual/required ratio.

## Methods

### Study description

**Figure 1:**
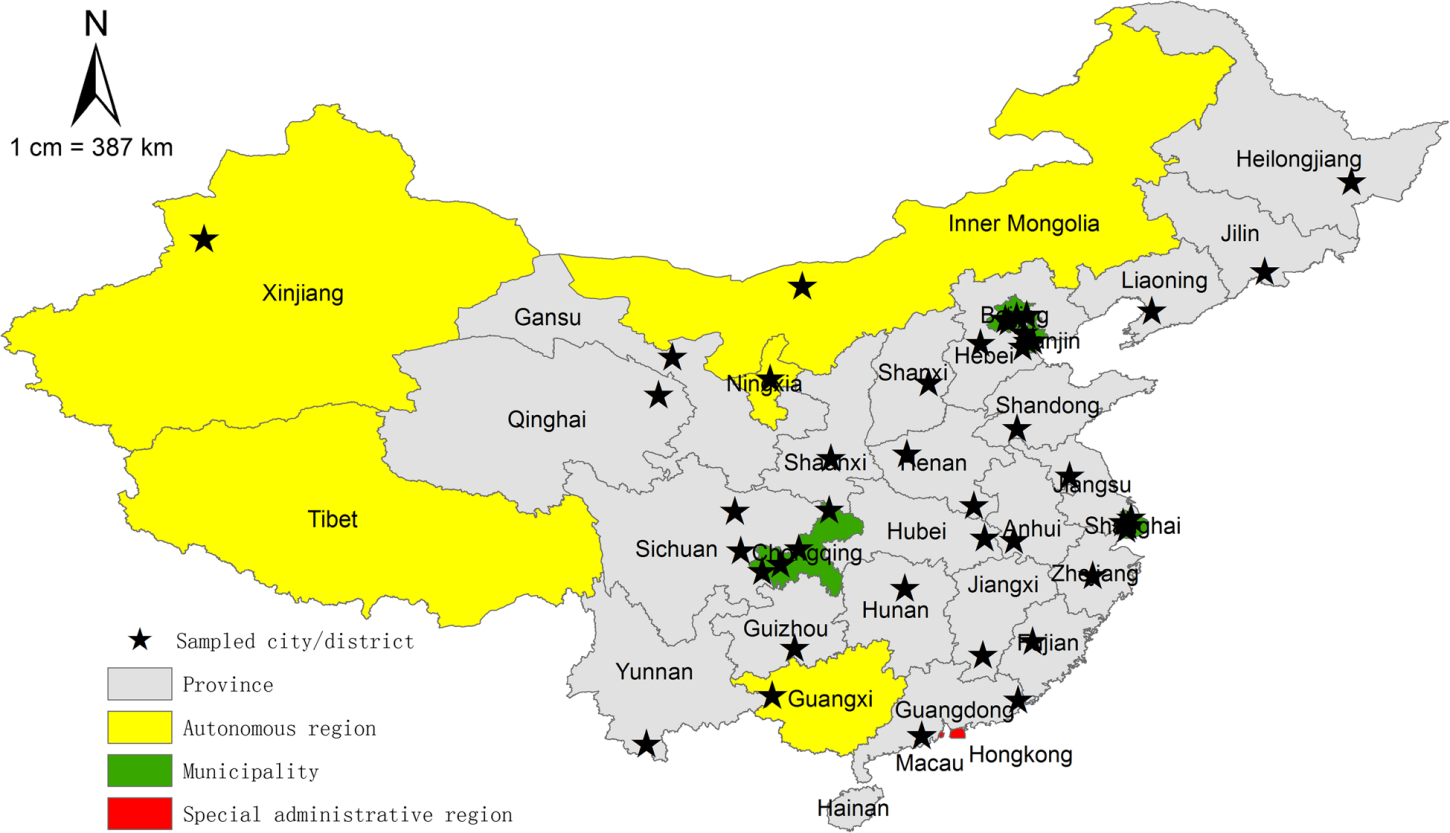
**Map of China showing the 22 provinces, 5 autonomous regions, 4 municipalities and selected samples.**

This study is a national institution-based sampling survey with formal administrative approval from the national maternal and child health annual report office. It was also supported by the National Health and Family Planning Commission of the People’s Republic of China (the former Chinese Ministry of Health until 2013) and it had been publicly announced in the Chinese Center for Disease Control and Prevention [23] and Xi’an Technology Resources Market [24]. In China, there is a hierarchical administrative health network, the top-down sequence is province level, prefectural level, county level, township level, village level [9]. In this study, health institutions at prefectural level were the sampling units as sampling at prefectural level could cover all levels and types of medical and health care units. We applied the following selecting procedure:

In the 22 provinces and 5 autonomous regions in China (Figure 1), 28 districts/cities had been sampled from the 332 municipality prefectural districts/cities using random clustering sampling, in which the randomly selected districts were considered as clusters and all the health care facilities in them were included in the study. In addition, two urban districts and two rural counties were drawn respectively from the four municipalities (Beijing, Shanghai, Tianjin and Chongqing) in China using random clustering sampling. This sampling design did not aim for provincial level estimates. The sample cities were from all province level administrative divisions in China except for Tibet autonomous region, Hainan province (there are only a few cities) and the two special administrative regions (Hong Kong and Macau). A total of 5,168 medical and health care institutions, which were all the institutions that actual provided maternal and child health in the selected urban districts, rural counties and districts/cities. A structured questionnaire for collecting data on human resources was developed and piloted. The questionnaire was then sent to all participating health institutions. All data was anonymized and we were granted permission to use and analyze the data by the participating institutions.

Since our research focus was the level of WCH resources we excluded all other auxiliary maternal and child health workforce personnel (including personnel involved in health education, training and supervision, management, project/research/education and statistics). For our study WCH professionals are health workers with legal medical certificates that provided women and children’s curative and preventive healthcare. All other workers who did not provide WCH services and workers who did not hold legal medical certificates were not counted. In addition to the number of WCH professionals, we also measured the annual WCH service workload of an institution by service types.

### Estimating the required number of WCH professionals

To estimate the required WCH workforce, we firstly calculated the time that a WCH professional could spend at work annually (after deducting weekends, public holidays, annual leave, training and sick leave). Then, the time norm for conducting each maternal or child related health service was set by an expert review of WCH experts from Beijing (25 experts), Tianjin (15 experts) and Gansu (35 experts) using the Delphi method. The time norm was the average time a health professional needed to perform one activity, counting from the start of this activity to the start of a next similar activity. Thirdly, we multiplied the service workload (of 2010) by the time norm of WCH services and got the total required time. Finally, we got the required number of WCH professionals by dividing the total required time by the time devoted by one health professional annually. Note that when estimating the required workforce, we assumed that the WCH professionals are well-trained, skilled, motivated and full-time workers.

### Statistical analysis

All the data was calculated and analyzed using SPSS 13.0. In order to get the actual number of WCH professionals and WCH workload of the whole country in 2010, we weighted the workload data based on the proportion of sampled districts/cities from each area before calculation. As a result, the weights were 4 for Beijing and Tianjin, 4.5 for Shanghai, 10 for Chongqing and 11 for every other city (see Appendix 1 for more details). To evaluate the situation of Chinese WCH workforce in 2010 we calculated the difference between the actual and required WCH professionals and the ratios of the actual-to-required number of staff. Data was grouped into three geographic regions: East, Central and West China, as defined by the Chinese Government. This categorization is both geographical and economical as the three different groups represent different levels of economic development with the East areas being the most developed and the West areas the least. The East provinces are Beijing, Tianjin, Hebei, Liaoning, Shanghai, Jiangsu, Zhejiang, Fujian, Shandong, Guangdong and Hainan; the Central provinces are Shanxi, Jilin, Heilongjiang, Anhui, Jiangxi, Henan, Hubei and Hunan and the West provinces are Inner Mongolia, Guangxi, Shaanxi, Gansu, Qinghai, Ningxia, Xinjiang, Sichuan, Chongqing, Guizhou, Yunnan and Tibet [25].

## Results

In total, there were 515,778 health professionals providing WCH services in China in 2010 (Table 1). There were more women’s than children’s health professionals in all three regions (East, Central and West of China), accounting for around 60% and 40% respectively. The services provided by the WCH professionals are presented in Tables 2 and 3. For each type of service, the corresponding annual workload was calculated and, outpatient and emergency services occupied the largest part of routine services, both in women and children departments (Tables 2 and 3). Workloads of most women’s services were larger in East than Central and West areas, except for the departments of gynecology/obstetrics outpatient and emergency and of vaginal delivery. For children’s health, workloads in East regions were also larger than that in Central and West regions. However, time spent for professional advising and consulting in East regions was very low, accounting for only 0.1% of the total.

**Table 1:** **Women and child health professionals in East, Central and West China**

**Region**	**Women health professionals**	**Child health professionals**	**Total**
East	110,535 (63.4%)	63,895 (36.6%)	174,430 (33.8%)
Central	112,805 (62.8%)	66,759 (37.2%)	179,564 (34.8%)
West	92,042 (56.9%)	69,742 (43.1%)	161,784 (31.4%)
**Total**	**315,382 (61.1%)**	**200,396 (38.9%)**	**515,778**

**Table 2:** **Service time norms, workloads and required numbers of women’s health professionals in East, Central and West areas of China**

**Service type**	**Time norm (minutes)**	**East**		**Central**		**West**		**Total**	
		**Workload (times)**	**Required professionals**	**Workload (times)**	**Required professionals**	**Workload (times)**	**Required professionals**	**Workload (times)**	**Required professionals**
Gynecology/obstetrics outpatient and emergency	10	100987247	10929 (34.8%)	86037768	9311 (29.6%)	103410760	11192 (35.6%)	290435775	31432
Professional advising and consulting	15	21602719	3507 (31.8%)	22272702	3616 (32.8%)	24034874	3902 (35.4%)	67910295	11024
Premarital/preconception health checkup	10	5235813	567 (57.7%)	1775664	192 (19.6%)	2064646	223 (22.7%)	9076123	982
Family planning operation	30	5761371	1871 (37.7%)	3998898	1298 (26.2%)	5518310	1792 (36.1%)	15278579	4961
Antenatal examination	10	36901587	3994 (44.9%)	23501165	2543 (28.6%)	21787293	2358 (26.5%)	82190044	8895
Antenatal screening	10	5603484	606 (51.8%)	2002033	217 (18.5%)	3204861	347 (29.7%)	10810378	1170
Antenatal diagnosis	10	2159626	234 (41.2%)	1650572	179 (31.4%)	1439907	156 (27.5%)	5250105	568
Vaginal delivery	420	3232860	14695 (33.5%)	3705185	16842 (38.4%)	2705629	12298 (28.1%)	9643674	43835
Caesarean section	120	2585493	3358 (42.0%)	2061290	2677 (33.5%)	1513121	1965 (24.6%)	6159904	8000
Postpartum visit within hospital	10	2693203	291 (45.8%)	1570602	170 (26.7%)	1608929	174 (27.4%)	5872734	636
Postpartum visit outside hospital	60	7691330	4994 (38.1%)	6682810	4339 (33.1%)	5820741	3780 (28.8%)	20194881	13114
Gynecological disorder screening	10	32006400	3464 41.0%)	24094577	2608 (30.8%)	22033974	2385 (28.2%)	78134951	8456
**Total**			**48510 (36.5%)**		**43992 (33.1%)**		**40571 (30.5%)**		**133073**

**Table 3:** **Service time norms, workloads and required numbers of children’s health professionals in East, Central and West areas of China**

**Service type**	**Time norm (minutes)**	**East**		**Central**		**West**		**Total**	
		**Workload (times)**	**Required professionals**	**Workload (times)**	**Required professionals**	**Workload (times)**	**Required professionals**	**Workload (times)**	**Required professionals**
Pediatrics/neonatology outpatient and emergency	10	127191936	13765 (46.6%)	65419486	7080 (24.0%)	80100760	8669 (29.4%)	272712182	29514
Pediatrics/neonatology outpatient infusion	10	81141955	8782 (47.4%)	48337598	5231 (28.4%)	40429969	4376 (23.8%)	169909522	18388
Professional advising and consulting	20	17148	4 (0.1%)	8167284	1768 (35.8%0	14638573	3169 (64.1%)	22823005	4940
Newborn visit within hospital	10	1978733	214 (40.5%)	1591623	172 (32.6%)	1306245	141 (26.7%)	4876601	528
Newborn visit outside hospital	60	7739557	5026 (31.6%)	6800395	4416 (27.7%)	9981032	6481 (40.7%)	24520983	15923
Newborn PKU screening	10	7739338	838 (49.9%)	2905288	314 (18.7%)	4871043	527 (31.4%)	15515669	1679
Newborn CH screening	10	21605422	2338 (74.2%)	2706826	293 (9.3%)	4809210	520 (16.5%)	29121458	3152
Newborn hearing screening	10	5133717	556 (62.1%)	1455102	157 (17.5%)	1691030	183 (20.4%)	8279849	896
Other neonatal screening	15	2501788	406 (92.7%)	132649	22 (5.0%)	62777	10 (2.3%)	2697214	438
Child health check-up	20	40473786	8761 (41.7%)	31236117	6761 (32.2%)	25357409	5489 (26.1%)	97067312	21010
Children’s dental care	15	9660676	1568 (47.5%)	3882352	630 (19.1%)	6801127	1104 (33.4%)	20344155	3303
Children’s eye care	10	9043461	979 (33.9%)	3464198	375 (13.0%)	14194846	1536 (53.1%)	26702505	2890
Children’s hearing care	10	6720916	727 (52.4%)	2832567	307 (22.1%)	3272571	354 (25.5%)	12826054	1388
Vaccines for children	10	113444700	12278 (41.4%)	85858080	9292 (31.4%)	74457362	8058 (27.2%)	273760142	29628
**Total**			**56241 (42.1%)**		**36818 (27.4%)**		**40618 (30.4%)**		**133677**

*Abbreviation: PKU* phenylketonuria, *CH* congenital hypothyroidism.

### The required number of WCH professionals

After accounting for weekends, public holidays, annual leave, sick leave and training/workshop days, there are 220 working days for a health professional. There are 7 working hours or 420 working minutes per day after accounting for one hour/day spent in average in other non-patient related activities (Delphi exercise). Therefore, each WCH professional devotes a maximum of 92,400 minutes every year in patient related tasks.

Time norm for each service was estimated by the Delphi exercise. We conducted face-to face expert interviews with WCH experts and adopted the median of each WCH service time as the time norm for this service/activity (Tables 2 and 3). Ten different services were included in family planning including intrauterine device insertion (25 minutes), intrauterine device removal (25 minutes), vasectomy (30 minutes), tubal ligation (35 minutes), vacuum aspiration abortion (30 minutes), dilatation and curettage for abortion (30 minutes), medical abortion (30 minutes), mid-trimester induced abortion (60 minutes), contraceptive implant (30 minutes) and contraceptive implant removal (30 minutes). We used the average time needed for each of the family planning services as the time norm for family planning related services (30 minutes).

We then multiplied the service workload with the corresponding time norm for calculating the required time for providing these services in 2010. Finally we estimated the number of required WCH professionals in the three Chinese regions by dividing the required time by the total working time one WCH professional could provide per year. For women’s health professionals, the corresponding numbers of health professionals were 133,073, 48,510, 43,992, 40,571 for China, East, Central and West regions respectively (Table 2). Similarly, for children’s health professionals, the estimated human resources needs were 56,241 in the East regions, 36,818 in the Central regions, 40,618 in the West regions and 133,677 in China (Table 3).

The difference between the actual and required professionals and the actual/required ratio for women’s health and children’s health was calculated for China and the three regions (Table 4). For the whole country, the number of WCH professionals’ surplus was 249,028 and the actual/required ratio was 1.93, which suggests that in 2010, the WCH professionals in China were sufficient to provide the WCH services at that time. Our data suggests that the WCH professionals’ surplus was mainly contributed by women’s health professionals’ excess (Table 4). Furthermore, in East regions, the actual/required ratio for children’s health was 1.14, suggesting that the manpower providing basic child health care was almost saturated.

**Table 4:** **Comparison between the actual and required WCH professionals in China and in East, Central and West regions**

**Region**	**Women’s health**				**Children’s health**				**Total**			
	**Actual**	**Required**	**Excess/gap**	**Actual/required ratio**	**Actual**	**Required**	**Excess/gap**	**Actual/required ratio**	**Actual**	**Required**	**Excess/gap**	**Actual/required ratio**
East	110535	48510	62025	2.28	63895	56241	7654	1.14	174430	104751	69679	1.67
Central	112805	43992	68813	2.56	66759	36818	29941	1.81	179564	80810	98754	2.22
West	92042	40571	51471	2.27	69742	40618	29124	1.72	161784	81189	80595	1.99
**Total**	315382	133073	182309	2.37	200396	133677	66719	1.50	515778	266750	249028	1.93

## Discussion

This study is the first nationwide WCH human resources investigation in China. We presented the actual and required number of WCH professionals by applying the utilization method. This is useful to offer workload-based human resources estimation for national, regional and local health policy-making. By calculating the difference between the actual and required number of WCH professionals and the actual/required ratio, which were referenced from the famous WISN method, it suggests that the actual WCH workforces were sufficient or even affluent in providing the WCH services in 2010, which is similar with the situation of China’s health workforce [12]. This phenomenon may be related with the massive expansion of medical education in China since 1998. The surplus of women’s health care professionals was much larger than the surplus of health care professionals providing children’s health care and in particular the children’s health professionals’ workload pressure was highest in East regions.

The samples selected for this survey were for the purpose of estimating WCH workforce at the national level. Since our samples cover a relative large range of areas and have relatively symmetrical distribution, we believe that our samples have national representativeness (Figure 1). Furthermore, the four municipalities were sampled separately, so that the corresponding samples could represent the four municipal cities respectively. However for the provinces and autonomous districts, we cannot be certain about the sampling representativeness and therefore we cannot use the samples to estimate the MCH workforces in each of the provinces or autonomous districts.

A significant advantage of this study is that the calculation of the required number of health staff was based on actual WCH service categories together with their corresponding workloads. Therefore, we can also know the contents and quality of the available WCH services. The outpatient and emergency departments were the busiest service sectors in the whole nation and the three regions, both for women’s and children’s health. The East region, which includes the most developed areas in China, had the largest workload in most WCH services when compared to the other two regions. For example newborn screenings is an important service focus in East China, but professional advising and consulting for children was not and this information may be useful for policy-makers for future health work planning.

When making time norm for each service type, it’s hard for a large developing country like China, because they can’t be attributed to a specific health professional nor they turn into a standardize maternity path (service target) to be granted to each pregnant mother or to each newborn across the whole, this is a limitation of our study. So we consulted WCH experts from different cities and different facilities in order to make the norms’ representativeness better. These norms could also be further used in China as a tool for evaluating WCH service performance at a national level or estimating the required WCH human resources in a different year. Also, for the actual number of WCH professionals, we acquired reliable routine data from each health institution and all these data resources allowed a realistic and objective measure of service utilization. Although it is possible to estimate the workload and then required number of professionals in different services, it is not possible to count the actual number of WCH professionals in each service type, since in the real world, one WCH professional may be occupied in many different services in the same time. Therefore, we could only make comparison between the actual and required number of WCH professionals in large scales, such as at regional and national level.

According to the development plan for women and children (2011–2020), the Chinese government put an emphasis on expanding the WCH human resources [6], however, the results of this study suggest that the WCH professionals in China were sufficient in 2010. For development and realizing universal health coverage in China, we do believe that expanding the WCH human resources might be necessary in the future and there is also potential for improvement by providing better services, but the priority should be improving the quality of WCH services rather than just adding new workforce.

## Conclusions

The WCH professionals in China were sufficient for the actual workload in 2010, there were still lots of potential capacities to provide better WCH services, especially for women. On the national level, kinds of strategies should be taken to improve the quality of WCH professionals or their working motivation.

## Appendix 1

### Weighting Method

Because the sampling unit is Cities at prefectural level (including prefectural cities in province and districts in municipalities), so the weights are mainly based on the proportion of sampled districts/cities:

For Beijing, there are 16 districts, and we selected 4, so the weight was 16/4 = 4.

For Tianjin, there are 16 districts, and we selected 4, so the weight was 16/4 = 4.

For Shanghai, there are 15 districts, and we selected 4, so the weight was 18/4 = 4.5.

For Chongqing, there are 15 districts, and we selected 4, so the weight was 40/4 = 10.

For all provinces and autonomous regions, there are total 332 prefectural level cities, from which we select 28, so the weight should be 332/28 = 11.8, but we decreased it to 11 by experts consulting and research experience.

Finally, by using the weights, the results turned out to be good, the sum of the weighted populations in the sample districts/cities was very near (with only 5.7% errors) to the actual population number (China census 2010).

## Abbreviations

WCH: Women and children’s health
MDG: Millennium Development Goal
WISN: Workload indicators of staffing need
